# In Vitro Studies on Zinc Binding and Buffering by Intestinal Mucins

**DOI:** 10.3390/ijms19092662

**Published:** 2018-09-07

**Authors:** Maria Maares, Claudia Keil, Jenny Koza, Sophia Straubing, Tanja Schwerdtle, Hajo Haase

**Affiliations:** 1 Department of Food Chemistry and Toxicology, Berlin Institute of Technology, Gustav-Meyer-Allee 25, D-13355 Berlin, Germany; maares@tu-berlin.de (M.M.); c.keil@tu-berlin.de (C.K.); jenny.koza@gmx.de (J.K.); Sophia.Straubing93@gmx.de (S.S.); 2 Institute of Nutritional Science, University of Potsdam, Arthur-Scheunert-Allee 114-116, 14558 Nuthetal, Germany; taschwer@uni-potsdam.de; 3 TraceAge-DFG Research Unit on Interactions of essential trace elements in healthy and diseased elderly, Potsdam-Berlin-Jena, Germany

**Keywords:** intestinal zinc resorption, zinc binding, mucus layer, intestinal mucins, in vitro intestinal model, goblet cells, Caco-2/HT-29-MTX-model

## Abstract

The investigation of luminal factors influencing zinc availability and accessibility in the intestine is of great interest when analyzing parameters regulating intestinal zinc resorption. Of note, intestinal mucins were suggested to play a beneficial role in the luminal availability of zinc. Their exact zinc binding properties, however, remain unknown and the impact of these glycoproteins on human intestinal zinc resorption has not been investigated in detail. Thus, the aim of this study is to elucidate the impact of intestinal mucins on luminal uptake of zinc into enterocytes and its transfer into the blood. In the present study, in vitro zinc binding properties of mucins were analyzed using commercially available porcine mucins and secreted mucins of the goblet cell line HT-29-MTX. The molecular zinc binding capacity and average zinc binding affinity of these glycoproteins demonstrates that mucins contain multiple zinc-binding sites with biologically relevant affinity within one mucin molecule. Zinc uptake into the enterocyte cell line Caco-2 was impaired by zinc-depleted mucins. Yet this does not represent their form in the intestinal lumen in vivo under zinc adequate conditions. In fact, zinc-uptake studies into enterocytes in the presence of mucins with differing degree of zinc saturation revealed zinc buffering by these glycoproteins, indicating that mucin-bound zinc is still available for the cells. Finally, the impact of mucins on zinc resorption using three-dimensional cultures was studied comparing the zinc transfer of a Caco-2/HT-29-MTX co-culture and conventional Caco-2 monoculture. Here, the mucin secreting co-cultures yielded higher fractional zinc resorption and elevated zinc transport rates, suggesting that intestinal mucins facilitate the zinc uptake into enterocytes and act as a zinc delivery system for the intestinal epithelium.

## 1. Introduction

The essential trace element zinc is predominantly resorbed in the small intestine, where it is absorbed by enterocytes and transported into the blood stream, primarily mediated by the apically located Zrt-, Irt-like transporter (ZIP)-4 and the basolateral zinc exporter (ZnT)-1 [1]. These two transporters are complemented by the basolateral transporter ZIP-5, importing zinc from the blood into the enterocytes, and the apical transporter ZnT-5, which exports zinc back into the intestinal lumen [1]. However, despite ongoing research, the molecular mechanisms regulating zinc absorption are not yet fully elucidated. Human intestinal zinc resorption was shown to be a regulated process, as the fractional zinc resorption varies between 20–60%, generally decreasing with elevated zinc intake [2]. The amount of absorbed zinc is not only influenced by oral zinc intake, but particularly depends on its accessibility in the intestine, which is strongly influenced by food components such as phytate, impairing the intestinal zinc availability. Additionally, amino acids and several trace elements were reported to impact enterocytes’ zinc uptake [3]. Notably, the intestinal availability of trace elements in general does not exclusively depend on food components, but is also influenced by the intestinal mucus layer. Specifically, the mucus was shown to bind ions such as iron, lead, and zinc preventing their hydroxypolymerisation at intestinal pH and increasing their solubility and availability for the intestinal epithelium [4,5,6]. It has been suggested that the affinity of mucins for metals increases from M^+^ < M^2+^ < M^3+^ leading to a competitive binding to the glycoproteins and consequentially influencing their bioavailability [5,7,8]. In fact, while the impact of mucins for iron resorption was investigated in detail, there is evidence that the mucus layer might also be important for zinc uptake by the human intestinal mucosa, as zinc binding by mucins was observed in animal studies [4,5,9,10]. Nevertheless, the detailed role of the intestinal mucus on human zinc absorption has not yet been investigated.

Mucus, synthesized and secreted by goblet cells, covers the entire gastrointestinal tract protecting the underlying epithelium against the luminal content, and plays an essential role in nutrition and health [11]. While a single loosely bound mucus layer supports the resorption of nutrients in the small intestine, this physical barrier is extended by an additional adherent mucus layer in the stomach and colon [11]. The gastrointestinal mucus is mainly constituted of water, ions, lipids and 5–10% highly glycosylated proteins: the mucins. These proteins maintain their macromolecular network-like structure by being largely composed of Serin, Threonin and Prolin tandem-repeats and O-linked oligosaccharides, as well as of fewer *O*-glycosylated cysteine-rich regions (recently reviewed in [12,13]). Thus, the intestinal epithelium does not only consist of enterocytes, absorbing the nutrients from the luminal content, but contains a variety of other cell types of which goblet cells are the most abundant, constantly secreting mucins into the lumen [14]. 

In vitro intestinal models provide a standardized and easy platform to analyze the bioavailability of nutrients, such as trace elements, as well as transport kinetics [15], offering a promising tool to illuminate distinct molecular aspects of intestinal zinc resorption. Not only are changes in cellular zinc tracked by using inductively-coupled plasma mass spectrometry (ICP-MS) and flame atomic absorption spectrometry (FAAS), but the application of low molecular weight sensors as an approach to measure zinc uptake [16,17,18] gained importance to determine small changes in the intracellular zinc pool [19]. These models always need to resemble the in vivo situation, not only concerning buffer and medium constituents, but also cellular composition. Until now, in vitro studies on intestinal zinc uptake were mainly conducted using the Caco-2 model, which was already shown to express the main intestinal zinc transporters [17]. This cell line is very well characterized and differentiates into a cell monolayer morphologically and functionally representing the enterocytes in vivo [20]. Some disadvantages of this in vitro cell model concerning overexpression of the Pgp-protein and lack of a mucus layer were improved by introducing the Caco-2/HT-29-MTX co-culture [21,22]. This well characterized co-culture of Caco-2 cells and the goblet cell line HT-29-MTX [22,23] was shown to be covered by mucus with a thickness of at least 2–10 µm after fixation [21] and has already been used to investigate the role of mucins on bacterial adhesion [24] as well as on the resorption of nutrients [21,25,26]. Furthermore, this co-culture was recently applied by our group to study the impact of a basolateral zinc acceptor on zinc resorption [17]. 

The aim of this study is to examine the role of intestinal mucins for zinc resorption. Herein, zinc binding properties of these glycoproteins and their zinc affinity are investigated in cell-free measurements as well as in the presence of intestinal cells to clarify the role of the mucus layer on zinc uptake. Finally, zinc transport was measured comparing a conventional Caco-2 monoculture and the Caco-2/HT-29-MTX co-culture to investigate the impact of mucins on the actual zinc transfer.

## 2. Results

### 2.1. Zinc Binding by Intestinal Mucins

**Figure 1:**
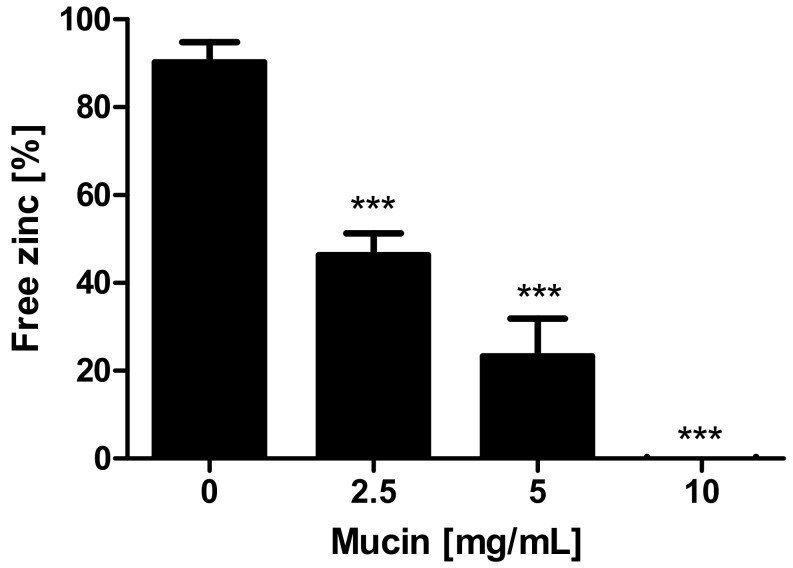
Effect of mucins on zinc availability for 4-(2-pyridylazo)resorcinol (PAR). Effect of mucins on zinc availability for PAR is shown as free zinc relative to the initially added zinc concentration. Different concentrations of zinc-depleted porcine mucin were incubated with 8 μM zinc and free zinc was analyzed using the colorimetric zinc chelator PAR. Data are presented as means + standard deviation (SD) of at least three independent experiments. Significant differences to the control are indicated (*** *p* < 0.001; one-way analysis of variance (ANOVA) with Dunnett’s multiple comparison test).

First, the property of mucins to bind and release zinc was investigated using the zinc-chelating chromophore 4-(2-pyridylazo)resorcinol (PAR) (additional spectrophotometric titration of the zinc-(PAR)_2_-complex in Supplementary Figure S1). Figure 1 shows a significant decrease of the free zinc concentration after applying 2.5–10 mg/mL zinc-depleted mucins (one-way analysis of variance (ANOVA) with Dunnett’s multiple comparison test; *p* < 0.001), indicating zinc binding by the gastrointestinal glycoproteins.

**Figure 2:**
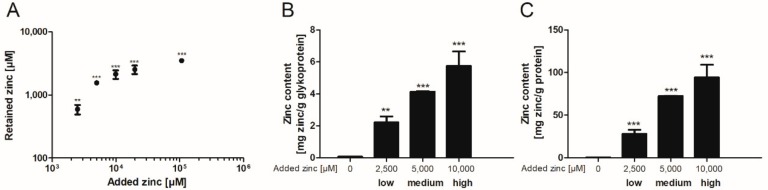
Zinc binding properties of gastrointestinal mucins. Different zinc concentrations were added to 25 mg/mL zinc-depleted porcine mucins and dialyzed against (Tris)-buffered saline (TBS) for 12 h. Subsequently the amount of zinc retained by binding to mucins was measured using flame atomic absorption spectrometry (FAAS) (**A**). Moreover, the zinc content of mucins after zinc loading is shown relative to the glycoprotein content measured by quantitative periodic acid Schiff (PAS)-assay (**B**), and relative to protein content of mucins measured by bicinchoninic acid (BCA)-assay (**C**). Data are shown as means ± SD of at least three independent experiments. Significant differences to the control are indicated (** *p* < 0.01; *** *p* < 0.001; one-way ANOVA with Dunnett’s multiple comparison test).

Subsequently, the binding capacity of these mucins was further investigated after dialysis of porcine mucins with different zinc concentrations for 12 h against Tris(hydroxymethyl)aminomethane (Tris)-buffered saline (TBS) (selection of the appropriate dialysis time in Supplementary Figure S2). Here, the zinc content of zinc-loaded mucins increased significantly compared to mucins without added zinc (Figure 2). The addition of 10,000 µM zinc before dialysis resulted in a beginning zinc-saturation of the glycoproteins (Figure 2A). These samples were defined as high zinc-loaded mucins. In total, three different degrees of zinc-loaded mucins were selected for further analysis: in addition to the high zinc-loaded mucins, also medium and low zinc-loaded mucins were dialyzed in the presence of 5000 µM or 2500 µM zinc, respectively. Next, zinc values were normalized to the total glycoprotein and protein content of the mucin samples after dialysis. These also depended on the amount of zinc present during dialysis, and maximum amounts of 5.7 mg zinc per g glycoprotein (Figure 2B) and of 94.2 mg per g protein (Figure 2C) were determined for the high zinc-loaded mucins.

**Figure 3:**
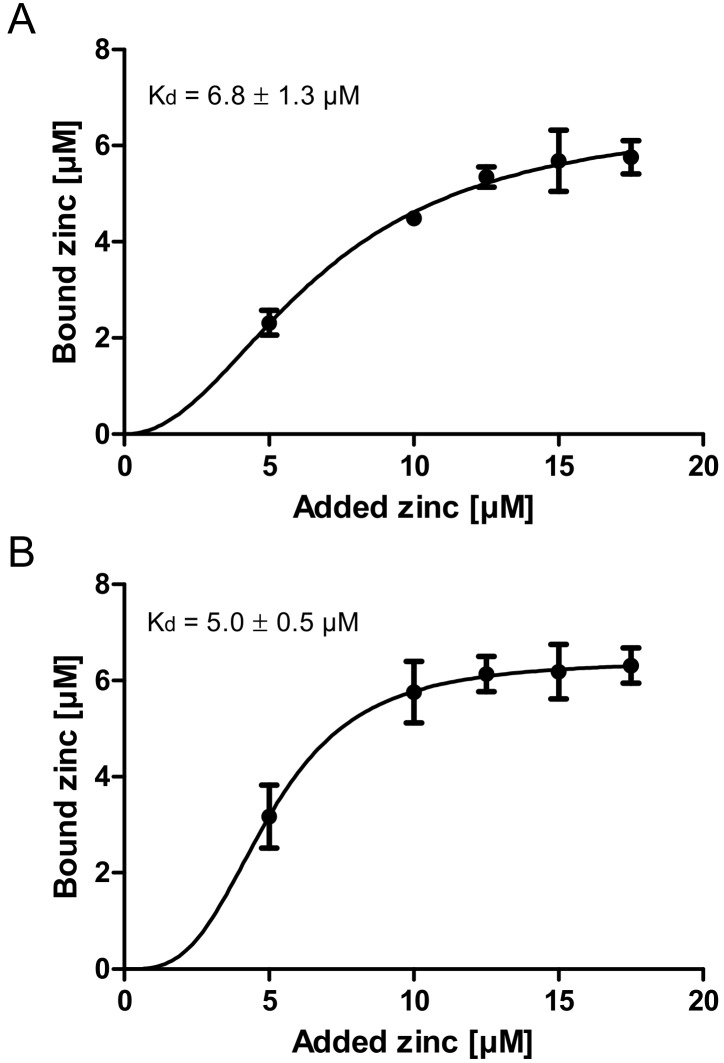
Zinc binding affinity of gastrointestinal mucins. Shown are zinc binding affinities of two different zinc-depleted mucins analyzed with the chromophore Zincon. For this, 1 mg/mL porcine mucins (**A**) and mucins harvested from HT-29-MTX (**B**) were used. Data were analyzed with GraphPad Prism software version 5.01 (GraphPad Software Inc., San Diego, CA, USA) and a non-linear regression assuming a one site-specific binding with Hill slope as a function of the zinc concentration was applied to calculate the dissociation constants of the mucin-zinc-complex as indicated. Data are presented as means ± SD of three independent experiments.

Finally, the zinc binding affinity of zinc-depleted porcine mucin was analyzed using the colorimetric reagent 2-carboxy-2′-hydroxy-5′-sulfoformazylbenzene monosodium salt (zincon) (additional spectrophotometric titration in Supplementary Figure S3) and compared to the affinity of zinc-depleted mucins harvested from the goblet cell line HT-29-MTX (Figure 3A,B). This analysis yielded similar dissociation constants for the zinc-mucin complexes with 6.8 µM for porcine and 5 µM for HT-29-MTX mucin.

### 2.2. Role of Zinc Buffering by Mucins on Zinc Uptake into Goblet Cells

**Figure 4:**
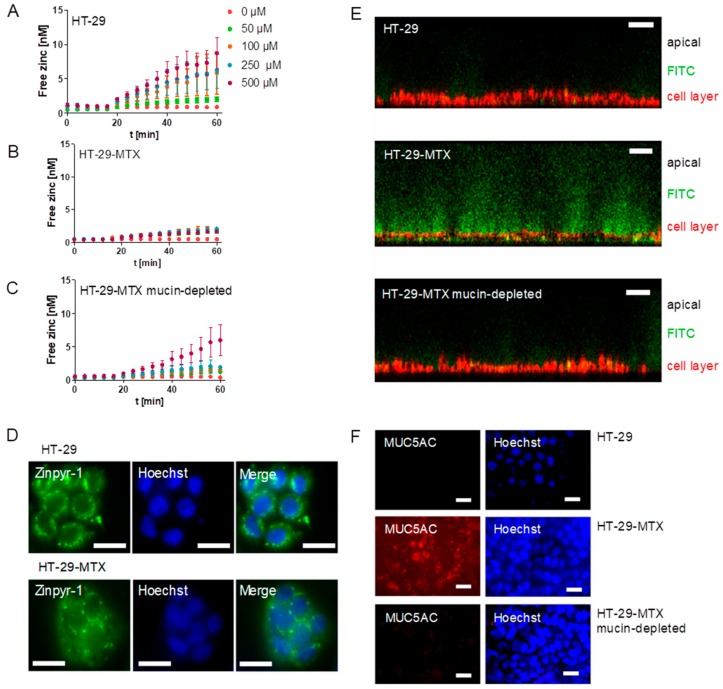
Effect of mucin depletion on zinc-resorption in HT-29-MTX. (**A**–**C**) Zinc uptake of the enterocytes HT-29 was analyzed with the fluorescent probe Zinpyr-1 (**A**) and compared to the zinc-uptake of the goblet cell line HT-29-MTX (**B**). Additionally, the effect of mucin depletion on zinc absorption of HT-29-MTX was analyzed after removing extracellular mucins using 10 mM *N*-acetylcysteine (**C**). Data are presented as means ± standard error of the mean (SEM) of three independent experiments. (**D**) Cellular distribution of Zinpyr-1 in HT-29 and HT-29-MTX cells together with nuclear staining using Hoechst was analyzed by fluorescence microscopy. Scale bar 20 µm. (**E**) Visualization of extracellular mucins with fluorescein isothiocyanate (FITC)-dextran was conducted using confocal laser scanning microscopy. Shown are z-stacks of HT-29, HT-29-MTX and HT-29-MTX without mucins after incubation with FITC dextran (green). The cell layer is stained with a cell membrane-tracker (red). Scale bar 50 µm. (**F**) Immunochemical detection of MUC5AC and nuclear staining using Hoechst using fluorescence microscopy in HT-29, regular HT-29-MTX and HT-29-MTX after removal of mucins. Scale bar 20 µm.

Next, the impact of mucins on zinc uptake by goblet cells was investigated. Zinc absorption of mucin-producing HT-29-MTX cells with or without mucin removal as well as the enterocyte cell line HT-29 was analyzed with the fluorescent low molecular weight zinc probe Zinpyr-1 and is depicted as the increase of intracellular free zinc (Figure 4A–C). Of note, the term “free” zinc is frequently used to describe the zinc pool that is complexed by small molecule ligands [27], which was already employed to investigate short-term zinc uptake in intestinal cells [16,17]. In detail, HT-29 showed a concentration-dependent zinc absorption (Figure 4A), while zinc uptake of the mucin-producing HT-29-MTX cells was very slight and concentration-independent (Figure 4B). When extracellular mucins were depleted with *N*-acetylcysteine (NAC), zinc uptake of HT-29-MTX cells increased (Figure 4C). Notably, analysis of the intracellular distribution of the fluorescent zinc sensor revealed a vesicular accumulation in both cell lines (Figure 4D). Extracellular mucins were visualized with the high molecular fluorescein isothiocyanate (FITC)-dextran 20 kDa (FD-20). It intercalates in the mucus layer due to its high molecular weight [28], and was analyzed using confocal laser scanning microscopy (CLSM) together with staining of the cell membrane (Figure 4E). Z-scans showed differences in the mucin thickness of HT-29 and HT-29-MTX, with HT-29 showing only slight FD-20-staining, whereas the HT-29-MTX goblet cells produced a thick extracellular mucin layer, which decreased visibly after mucin depletion. Moreover, mucin secretion of HT-29-MTX cells and its successful depletion with NAC was further investigated by immunofluorescent staining of the MUC5AC-apoprotein together with nuclear staining using Hoechst (Figure 4F). It shows a diffuse distribution of the MUC5AC-apoprotein over the cell layer of HT-29-MTX, whereas no MUC5AC-staining was observed for mucin-depleted HT-29-MTX and HT-29 cells.

### 2.3. Impact of Extracellular Mucins on Zinc Uptake into Enterocytes

**Figure 5:**
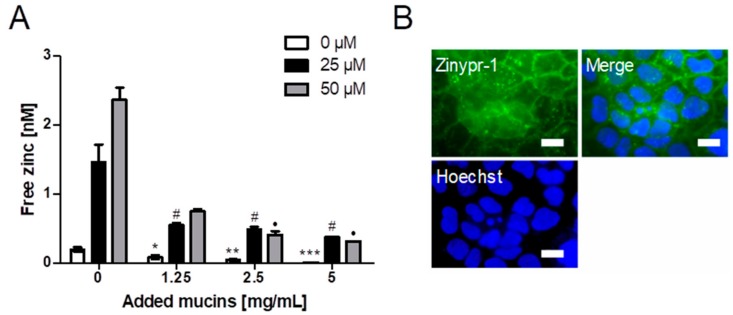
Impact of zinc-depleted mucins on zinc uptake by enterocytes. (**A**) Zinc uptake in Caco-2 cells after zinc incubation for 40 min in the presence of different concentrations of zinc-depleted porcine mucin is shown as the increase of free zinc using the fluorescent zinc probe Zinpyr-1. Data are shown as means + SD of at least three independent experiments. Significant differences from 0 mg/mL zinc-depleted porcine gastric mucin within one zinc concentration are indicated (*^,^^#,^^●^ *p* < 0.05; ** *p* < 0.01; *** *p* < 0.001, one-way ANOVA with Dunnett’s multiple comparison test). (**B**) Fluorescence microscopy showing the intracellular distribution of the zinc-dependent signal of the fluorescent probe Zinpyr-1 in Caco-2 cells together with nuclear staining using Hoechst. Scale bar 20 µm.

The impact of different mucin concentrations on short-term zinc uptake into the intestinal cell line Caco-2 was investigated with Zinpyr-1 (Figure 5A). Cellular free zinc is increasing after adding 25 and 50 µM zinc, respectively, but decreases significantly with added zinc-depleted porcine mucin (one-way ANOVA with the Bonferroni post hoc test: 1.25 mg/mL (25 µM): *p* < 0.05; 2.5 mg/mL (25 µM and 50 µM): *p* < 0.05; 5 mg/mL (25 µM and 50 µM): *p* < 0.05). Moreover, Figure 5B presents imaging of the cellular localization of Zinpyr-1 fluorescence in Caco-2 cells unveiling a predominantly vesicular distribution of the fluorescence. 

**Figure 6:**
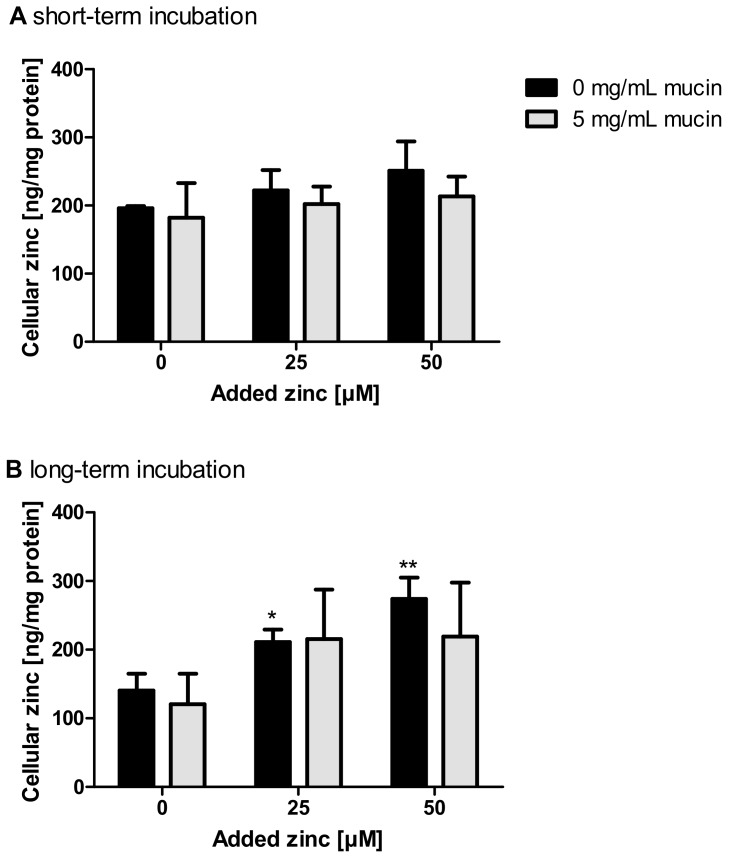
Impact of extracellular mucins on zinc uptake in Caco-2 cells measured with FAAS. The influence of extracellular addition of zinc-depleted mucins on the uptake of different zinc concentrations in enterocytes was investigated after incubation for 30 min (**A**) and 24 h (**B**). Cellular zinc content was analyzed using FAAS and is shown relative to cellular protein. Data are shown as means + SD of three independent experiments. Means significantly different from the untreated controls are indicated (* *p* < 0.05; ** *p* < 0.01; one-way ANOVA with Dunnett’s multiple comparison test).

Short-term zinc uptake in the presence of 5 mg/mL zinc-depleted porcine mucins was also investigated by FAAS, yielding no significant change of the cellular zinc content either with or without zinc-depleted mucins (Figure 6A). In comparison, long-term zinc absorption in the presence of 0 and 5 mg/mL zinc-depleted mucins, also conducted with FAAS, resulted in a significant increase of the cellular zinc content of Caco-2 cells after applying zinc without mucins (Figure 6B; one-way ANOVA with Dunnett’s multiple comparison test; 25 µM: *p* < 0.001; 50 µM: *p* < 0.01). The incubation of zinc together with 5 mg/mL mucins did not significantly change the cellular zinc content. 

**Figure 7:**
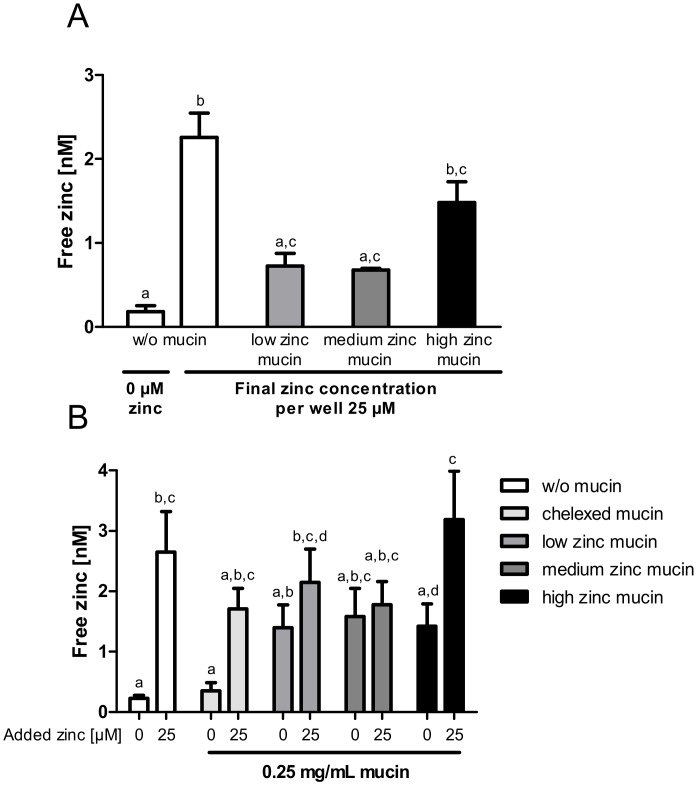
Effect of mucin zinc saturation on zinc uptake by enterocytes. The impact of the degree of zinc saturation of extracellular added mucins on zinc uptake in Caco-2 cells is shown by measuring the increase of free zinc using the fluorescent zinc probe Zinpyr-1. Porcine mucins were incubated with 2500 µM, 5000 µM and 10,000 µM zinc, resulting in mucins with differing zinc content (low, medium, high) and degree of zinc saturation. (**A**) Zinc absorption of Caco-2 cells after 40 min of treatment with 25 µM zinc alone or with zinc-loaded mucins, diluted to a final zinc concentration of 25 µM zinc (final mucin concentration: 1 mg/mL low zinc mucin, 0.46 mg/mL medium zinc mucin, 0.31 mg/mL high zinc mucin). (**B**) Zinc uptake after 40 min incubation with 0 µM or 25 µM zinc in the presence of 0 mg/mL or 0.25 mg/mL zinc-loaded or zinc-depleted mucins, respectively. Data are presented as means + SEM of at least three independent experiments. Significant differences were analyzed by repeated-measures ANOVA with the Bonferroni post hoc test. Bars sharing a letter (a, b, c, d) are not significantly different.

Next, the zinc buffering capacity of mucins and their zinc release into enterocytes was investigated in an experimental setting closer to the in vivo situation by using the aforementioned low, medium and high zinc-loaded porcine mucins. First, cellular zinc uptake from zinc-loaded mucins diluted to a final zinc concentration of 25 µM was compared to the absorption of 25 µM zinc without mucins (Figure 7A). While low and medium zinc-loaded mucins resulted in a comparable rise of intracellular free zinc, high zinc-loaded mucins caused a significant increase. However, they did not reach the levels of cellular zinc that were observed without mucins (Figure 7A).

To elaborate the influence of the zinc buffering capacity of mucins on cellular zinc uptake, the absorption of 25 µM zinc in the presence of 0.25 mg/mL zinc-loaded or zinc-depleted mucins was analyzed (Figure 7B). This was then compared to the uptake of 25 µM zinc without mucins. Here, intracellular free zinc increased significantly, whereas the presence of zinc-depleted mucins diminished the zinc uptake comparable to the results shown in Figure 5. Incubating the cells with 0.25 mg/mL zinc-loaded mucins (w/o additional zinc), a similar slight increase of free zinc regardless of the mucins’ remaining zinc-binding capacity and zinc content was detected. Adding zinc-loaded mucins and 25 µM zinc simultaneously to the cells yielded a significant rise of free zinc in the presence of high zinc-loaded mucins (repeated-measures ANOVA; high zinc-loaded mucins with 25 µM zinc compared to high zinc mucins with 0 µM: *p* < 0.05). This increase was similar to the absorption of 25 µM zinc in the absence of mucins. In contrast, low and medium zinc mucins, still not completely saturated with zinc, showed no additional zinc absorption in the presence of 25 µM zinc. 

### 2.4. Comparison of Zinc Resorption in Different Intestinal Cell Culture Models: The Role of Mucins 

Finally, the role of intestinal mucins on zinc resorption was analyzed comparing zinc transport by a Caco-2/HT-29-MTX co-culture with a Caco-2 monoculture. The integrity of the cell monolayers was monitored during the experiments by measuring the paracellular permeability for FD-20 and by detecting transepithelial electrical resistance (TEER) at the beginning and end of the experiments, revealing no impairment of both parameters during the resorption study (Supplementary Figure S4). The presence of goblet cells in the co-culture did not influence the permeability of the cell monolayer, as the transepithelial resistance of the co-cultures and Caco-2 monocultures did not differ significantly (co-cultures: 1146.7 ± 25.5 Ω·cm^2^; monocultures: 1288.9 ± 239.2 Ω·cm^2^) and the paracellular permeability was comparable to those measured in the absence of goblet cells (Supplementary Figure S4). 

**Figure 8:**
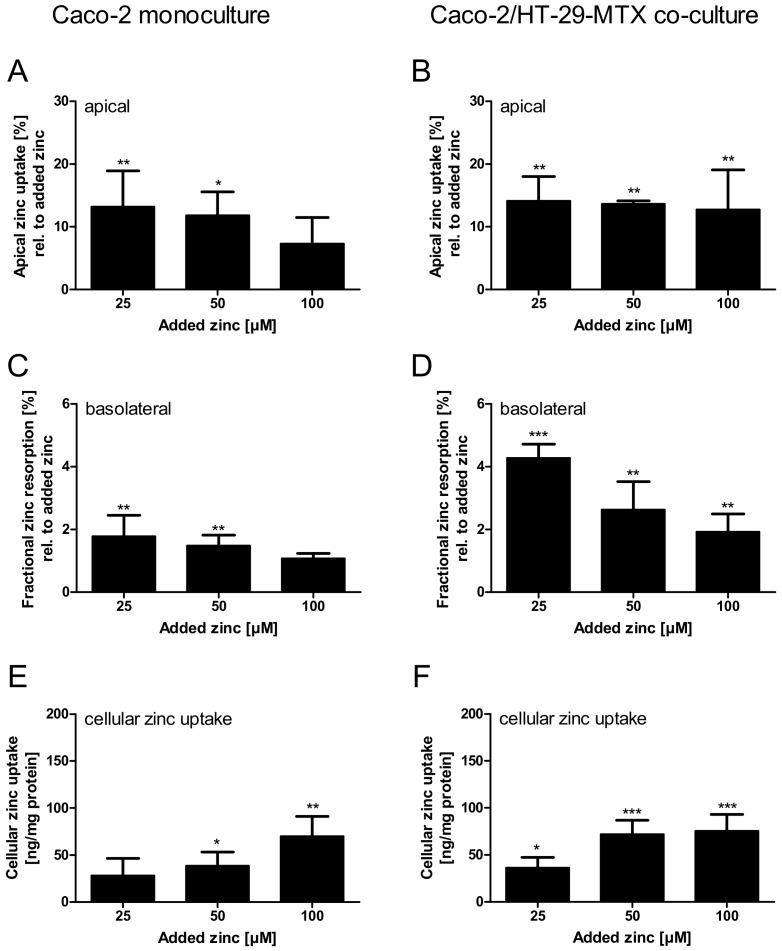
Comparison of zinc resorption in Caco-2 monocultures and Caco-2/HT-29-MTX co-cultures. Zinc transport studies were conducted using the enterocytes Caco-2 (**A**,**C**,**E**) and a co-culture of Caco-2 with the mucus-producing goblet cell line HT-29-MTX (**B**,**D**,**F**). Shown are the decrease of apical zinc (apical zinc uptake) (**A**,**B**) and fractional zinc resorption after 4 h incubation relative to the initially added amount of zinc (**C**,**D**). Moreover, cellular zinc uptake is shown relative to cellular protein content (**E**,**F**). Data are shown as means + SD of three independent experiments and means significantly different from the untreated controls are indicated (* *p* < 0.05; ** *p* < 0.01; *** *p* < 0.001; one-way ANOVA with Dunnett’s multiple comparison test). According to a two-way ANOVA with the Bonferroni post hoc test comparing the results within one added zinc concentration of the mono- and co-cultures there are significant differences regarding the fractional zinc resorption (25 µM: *p* < 0.001; 50 µM: *p* < 0.05), and zinc uptake (50 µM: *p* < 0.05).

Apical zinc uptake by the monoculture, relative to the initially applied zinc concentrations, was declining with increasing amounts of zinc. In contrast, the Caco-2/HT-29-MTX co-culture absorbed comparable amounts between 12.8% and 14.2% of all added zinc concentrations (Figure 8A,B). Regardless of the differences in the apical zinc uptake, the fractional zinc resorption into the basolateral compartment of both intestinal models declined inversely related to the initially added zinc. Yet, the fractional resorption was significantly higher in Caco-2/HT-29-MTX co-cultures (Figure 8C,D; two-way ANOVA with the Bonferroni post hoc test comparing the mono- and co-cultures: 25 µM: *p* < 0.001; 50 µM: *p* < 0.05). More precisely, fractional resorption by monocultures dropped from 1.6% to 0.9%, showing only slight concentration dependence, whereas resorption by co-cultures declined from 4.2% to 1.9% of the initially added zinc. Additionally, in both intestinal models the cellular zinc uptake increased with added zinc, yielding a significantly higher uptake after addition of 50 µM zinc by the co-culture (Figure 8E,F; two-way ANOVA with the Bonferroni *post hoc* test, *p* < 0.05).

**Figure 9:**
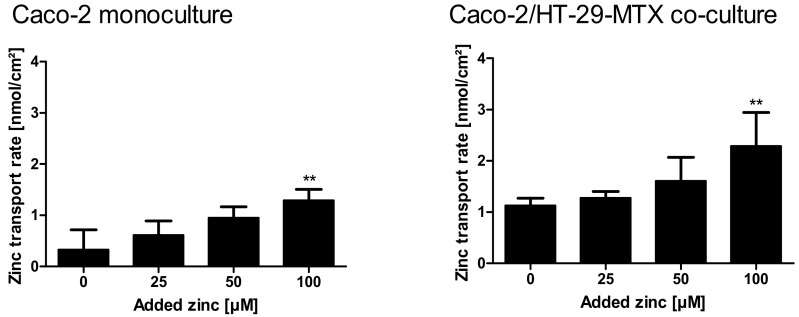
Zinc transport rates in Caco-2 monocultures and Caco-2/HT-29-MTX co-cultures. Zinc transport rates in nmol zinc per cm^2^ resorption area in mono- and co-cultures are displayed. Data are presented as means + SD of three independent experiments. Significant differences to control cells (0 µM zinc) are indicated (** *p* < 0.01; one-way ANOVA with Dunnett’s multiple comparison test). According to a two-way ANOVA with the Bonferroni post hoc test comparing the results within one added zinc concentration of the mono- and co-cultures, there is a significant difference between the zinc transport rate at 100 µM (*p* < 0.001).

Supplementary Table S1 summarizes detailed quantitative data of zinc uptake into the cells, cellular zinc content, and the amount of zinc transported to the basolateral compartment. Over all, the zinc transport study resulted in higher zinc transport rates for the mucin-producing co-culture (Figure 9A,B; monoculture: 0.3–1.29 nmol zinc/cm^2^; co-culture: 1.1–2.3 nmol zinc/cm^2^). In detail, according to a two-way ANOVA with the Bonferroni post hoc test comparing the results of the two intestinal models within one added zinc concentration, the co-culture resulted in a significantly higher zinc transport rate of initially added 100 µM (*p* < 0.05).

## 3. Discussion

Zinc binding by the mucus layer was already observed in animal studies [4,5,9,10], suggesting that mucins could play a role in human intestinal zinc absorption. However, little was known about the distinct zinc binding properties of these glycoproteins. The present study demonstrated that zinc binding is not only dependent on the amount of the available, or rather, free zinc concentration, but also on the zinc:mucin ratio. Assuming an approximate molecular mass of intestinal mucins of 2.5 MDa [29,30], the employed mucin concentrations between 2.5–10 mg/mL correspond to 1 µM–4 µM mucin, resulting in a 2–8 fold molar excess of zinc in the assay. When applying an 8-fold molar zinc excess, 50% of zinc was retained by the glycoproteins, declining to almost no free zinc at a molar zinc:mucin ratio of 2 (Figure 1). This suggests multiple zinc-binding sites within one mucin molecule with biologically relevant affinity. 

Notably, gastrointestinal mucins were able to decrease the amount of available zinc for the high affinity colorimetric reagent PAR (dissociation constant of the zinc-(PAR)_2_-complex 7.08 × 10^−13^ M^2^ [31]), indicating that at least some of the binding sites have high affinity for zinc. The coordination of metals by proteins strongly depends on the metal ion preference for particular electron donors [32]. Zinc tends to form stronger covalent binding to nitrogen and sulfur and weak complexes with oxygen [32]. The latter are highly present in mucins by carboxylate groups of the *O*-glycans [13]. Nitrogen and Sulfur are part of both human and porcine intestinal mucins containing *N*-acetyl groups (*N*-acetyl-galactosamine (GalNac), *N*-acetylneuraminic acid (NeuAc)) [33,34] and on average about 8–14% free thiols [35,36], possibly playing an important role in the zinc binding affinity of mucins. 

The zinc binding capacity of mucins was further investigated by dialysis of pig gastric mucins against different zinc concentrations, resulting in low, medium and high zinc-loaded mucins (Figure 2). According to the present study, mucins have an average molar zinc binding capacity of about 200, indicating a multiplicity of zinc-binding sites within one mucin molecule, possibly providing a broad spectrum of different binding affinities. Mucins are highly glycosylated proteins, consisting of approximately 80% carbohydrates [11,13]. Thus, the total amount of zinc bound per g protein is one order of magnitude higher than per g glycoprotein (Figure 2B,C). These results are comparable to those obtained by Quarterman et al. analyzing zinc binding of porcine mucins at different pH levels, which led to a zinc content of 10 mg zinc per g mucin at pH 7.5 [4]. Furthermore, the binding constants of porcine gastric mucin and mucins obtained from the cell line HT-29-MTX are in good agreement with each other (Figure 3), and additionally are of the same magnitude as luminal zinc levels [37,38]. Given the high number of binding sites, they probably represent an average dissociation constant of the zinc/mucin complex, constituting a mixture of several binding sites with varying affinities.

Diet-derived luminal factors influence intestinal zinc bioavailability [39]. Together with physiological factors such as luminal fluid and mucus layer, these components represent the luminal matrix, which influences zinc speciation, consequently affecting its availability for enterocytes [4,18]. To illuminate the impact of apically present zinc-binding proteins on zinc uptake into enterocytes, a recent study of our group investigated the effect of albumin on zinc absorption. Albumin significantly reduced short-term zinc uptake measured with the low molecular weight sensor Zinpyr-1 [17]. By the same technique, the role of mucins in short-term zinc absorption was analyzed in the present study. The goblet cell line HT-29-MTX produces and secretes mucins covering the cell surface, as shown by qualitative analysis using immunochemical staining of the MUC5AC-apoprotein (Figure 4E), which is comparable to previous MUC5AC-stainings of HT-29-MTX mucins [40]. Yet, the impact of the mucus layer on their zinc uptake has not been investigated before. Zinc uptake of HT-29-MTX before and after mucin depletion was analyzed and compared to the intestinal absorptive cells HT-29. Overall, mucin depletion caused a FD-20- and MUC5AC staining almost similar to that of HT-29 cells, confirming a successful removal of extracellular mucins by N-acetylcysteine. Notably, short-term zinc uptake of HT-29-MTX was impaired by extracellular mucins, which indicates zinc binding and buffering by the glycoproteins produced by these cells. In Caco-2 cells, cellular zinc absorption also decreased significantly with elevated concentrations of zinc-depleted porcine mucins (Figure 5A), comparable to the impairment by albumin [17]. In addition to the analysis with the low molecular weight sensor Zinpyr-1, short-term zinc uptake by Caco-2 cells was also measured with FAAS resulting in no significant change of cellular zinc, either with or without mucins (Figure 6A). Only long-term zinc incubation of Caco-2 cells resulted in a significant increase of cellular zinc in the absence of mucins (Figure 6B). Consistent with previous findings, changes in intracellular zinc after short-term incubation of Caco-2 cells are probably too small compared to the cellular zinc content to be detected by FAAS [17]. 

From the uptake studies shown in Figure 5A and Figure 6 it could be concluded that mucins impair intestinal zinc availability. However, these porcine mucins were zinc-depleted, which is compulsory for investigating zinc-binding capacity and affinity, but does not represent the in vivo situation in the intestinal lumen under zinc adequate conditions [38,41]. This is corroborated by the fact that the commercially available porcine mucins contained considerable amounts of zinc before zinc-depletion with Chelex^®^ 100 Resin. Accordingly, the impact of zinc-loaded mucins on zinc uptake into enterocytes was examined using zinc-containing mucins (low, medium and high zinc-loaded mucins; Figure 7). Here, all three types of mucins seemed to release part of their zinc, resulting in an increase of cellular free zinc compared to control cells (Figure 7A). Interestingly, this release appeared to be largely independent of the mucins’ zinc content and their remaining zinc binding capacity, adding the same concentration of differentially loaded mucins to the cells resulted in similar zinc uptake (Figure 7B). These observations suggest that mucins buffer the cellular available zinc concentration in the lumen, still keeping it available for enterocytes. In animal studies, intestinal mucins were discussed to absorb zinc from the luminal content transferring it to the mucosa [9,10]. Zinc transfer to the intestinal cells occurred slower than the initial zinc binding by mucins [9]. In this manner, the intestinal mucus layer might lead to retention of luminal available zinc, possibly providing intestinal cells with zinc for extended periods of time after food intake. This implies that mucins act as a zinc delivery system from the lumen to the intestinal epithelium, which was already postulated for iron [5], and led to our hypothesis that zinc-saturated mucins might facilitate zinc delivery to enterocytes. In the present study, however, cellular zinc uptake in the presence of zinc-saturated mucins was similar, but not augmented, compared to the absence of mucins (Figure 7B). This discrepancy might be due to insufficient equivalence to the in vivo situation: first, the basolateral compartment, where the absorbed zinc can be exported, is lacking. Second, the mucins applied in this study were commercially available isolated porcine mucins, which might not be entirely comparable to the native mucins produced by goblet cells [42]. Although these mucins are often used as a standard model for the mucus layer to investigate characteristics of gastrointestinal mucins [43] and their role in intestinal metal uptake [28], they are also known to have weaker gel-forming abilities [44], possibly due to protease treatment during the isolation and purification process [42]. 

To overcome both issues, the impact of chemically unprocessed mucins on intestinal zinc resorption in a three-dimensional culture was investigated by transport studies in the mucin-producing Caco-2/HT-29-MTX co-culture, which were then compared to zinc transport in the absence of a mucus layer using conventional Caco-2 monocultures. Herein, the mucin-producing co-culture clearly yielded higher zinc absorption than the mucus-lacking monoculture (Figure 8). The Caco-2/HT-29-MTX model resulted in a stronger decrease of the apical zinc concentration, varying around 12.7–14.1% independent of the initially added zinc, as well as an elevated cellular zinc uptake (Figure 8E,F), indicating that the cellular zinc uptake is facilitated by the mucus layer. Indeed, this supports the aforementioned hypothesis that the mucus layer might act as a zinc delivery system for the intestinal epithelium. Moreover, the glycoproteins seemed to assist the zinc transfer across the intestinal epithelium as the fractional zinc resorption was significantly higher (1.8–2.4 fold higher) in the presence of mucin-producing goblet cells. Likewise, the zinc transport rate was elevated using co-cultures. Here, the basal zinc transport rate without the addition of exogenous zinc was already 3.8 fold higher (1.13 ng zinc/cm^2^ resorption area) than that of monocultures (0.3 ng zinc/cm^2^) (Figure 9), possibly due to resorption of the basal zinc levels of mucins. These basal zinc levels might originate from the cell culture medium (containing 3 µM zinc), and were incorporated by the mucins during the 21 days of cultivation of the cell model. Thus, the mucus-secreting co-culture not only absorbed more zinc from the apical compartment, but showed augmented zinc export to the basolateral side, supporting previous observations that intestinal mucins represent an important factor for intestinal zinc resorption [4,9,10]. 

The beneficial role of mucins for the resorption of other trace elements, such as iron, is already well documented [21,28,45]. Concerning the essential nutrient iron, the mucus layer not only mediates its availability for the intestinal epithelium by maintaining its solubility [5], but was also proposed to provide its delivery to the absorptive cells by the mucin-integrin mobilferrin pathway [45,46]. A similar mechanism might also be effective for zinc, as the present study indicates that mucins not only influence zinc uptake by increasing its luminal solubility, as discussed before [4], but rather promote zinc absorption by additionally acting as a zinc delivery system for the mucosa, which would be in good agreement with observations from animal studies [9,10].

Metal ion binding by mucins might have further implications for metal ion homeostasis. The impact of other trace elements on zinc resorption was previously investigated and is still a topic of ongoing research [3,47]. Mucins are not only involved in the mucosal uptake of single trace elements, but were also suggested to influence their bioavailability by competitively binding different metals [5]. Thus, in addition to competing for transport proteins, competition for binding sites in mucins could be another factor for the mutual interferences observed in intestinal trace element absorption. Furthermore, mucins not only support intestinal zinc absorption, but might also be involved in fecal zinc loss. A considerable amount of endogenous zinc is excreted with feces [2,48]. Even during extreme zinc deficiency, the fecal excretion of zinc can be observed, which is defined as the “obligatory fecal loss” [2]. The intestinal epithelium and the overlying mucus layer undergo cycles of renewal [49,50] resulting in a complete turnover of the mucins, which is said to occur much faster than that of the underlying mucosa [51]. Considering the amount of zinc bound to mucins, the fast renewal of the intestinal mucus layer might play an important role in the fecal zinc loss as the mucins are possibly excreted together with a remainder of tightly bound zinc.

On the one hand, gastrointestinal mucins are important for zinc absorption in the intestinal tract. On the other hand, zinc was also reported to be important for mucin synthesis, as the gene expression profile of different mucin proteins was shown to depend on zinc supply [52], and abdominal production as well as secretion of mucins were also decreased in zinc-deficient animals [53,54]. Thus, further investigations of the interplay of zinc with intestinal mucins and distinct molecular mechanisms of the mucosal delivery system are needed for a better understanding of their role in intestinal zinc uptake as well as gastrointestinal disorders.

## 4. Materials and Methods

### 4.1. Materials

CellMask™DeepRed (ThermoFisher Scientific, Waltham, MA, USA); Cy3^®^ goat anti rabbit IgG (Jackson ImmunoResearch, Dianova, Hamburg, Germany); Chelex^®^ 100 Resin (Bio-Rad, Hercules, CA, USA); Dulbecco’s modified Eagles medium (DMEM) (PAN-Biotech, Aidenbach, Germany); Hoechst 33258 (Sigma Aldrich, Munich, Germany); fluorescein isothiocyanate (FITC)-dextran 20 (TdB, Uppsala, Sweden); fetal calf serum (FCS) (CCPro, Oberdorla, Germany); mucin from porcine stomach Type II (Sigma Aldrich, Munich, Germany); PAR (Sigma Aldrich, Munich, Germany); Transwell inserts (Corning, New York, NY, USA); *N*,*N*,*N*′,*N*′-Tetrakis(2-pyridylmethyl)ethylenediamine (TPEN) (Sigma Aldrich, Munich, Germany); WillCo-dish^®^ glass bottom dish (WillCo, Amsterdam, The Netherlands); Zincon (Sigma Aldrich, Munich, Germany); ZnSO_4_·7H_2_O (Sigma Aldrich, Munich, Germany). All other chemicals were purchased from standard sources.

### 4.2. Cell Culture

Caco-2, HT-29-MTX and HT-29 cells were cultured at 37 °C, 5% CO_2_ and in a humidified atmosphere in DMEM, containing 10% FCS, 100 U/mL penicillin and 100 µg/mL streptomycin. Additionally, medium for HT-29-MTX contained 1% non-essential amino acids (NEAA). Media were changed every other day. Analysis of proper differentiation, morphology and barrier integrity of Caco-2 cells cultured in our lab into an enterocyte-like phenotype after 21 days, as shown before [20,55], was reported previously [56]. Caco-2, HT-29-MTX and HT-29 cells were obtained from European Collection of Cell Cultures (ECACC, Porton Down, UK).

### 4.3. 4-(2-Pyridylazo)Resorcinol (PAR) Assay

Zinc binding by mucins was investigated with the colorimetric reagent PAR. The assay was performed in TBS, containing 50 mM Tris(hydroxylmethyl)aminomethan and 150 mM NaCl, at pH 7.5. Stock solutions of 25 mg/mL porcine mucins were prepared in TBS and zinc-depleted using Chelex^®^ 100 Resin according to the manufacturer’s protocol (zinc content before depletion: 436 µg zinc/g mucin; zinc content after depletion: 16.2 µg zinc/g mucin). Different mucin concentrations were incubated with 8 µM ZnSO_4_·7H_2_O and 20 µM PAR (stock solution 25 mM in H_2_O) was added. Absorption of the zinc-(PAR)_2_-complex was measured at 485 nm using a well plate reader (M200, Tecan, Switzerland) and the effect of mucins on the availability of zinc for PAR was analysed using an external calibration of 0–10 µM zinc obtained by spectrophotometric titrations followed by linear regression analysis.

### 4.4. Zinc-Binding Capacity

The zinc-binding capacity of gastrointestinal mucins was investigated by dialysis. 25 mg/mL mucins were incubated with 0–100 mM ZnSO_4_·7H_2_O overnight and dialyzed against 1.5 L TBS for 6, 12 and 24 h. Finally, zinc concentrations were determined with FAAS using a Perkin Elmer AAnalyst800 (Perkin Elmer, Rodgau, Germany) and zinc-binding capacity was calculated relative to the zinc concentrations before dialysis. Furthermore, the amount of protein in the mucin samples after dialysis was analyzed using BCA-assay as described [57] and the glycoprotein content in the mucin samples was detected using the quantitative PAS-assay from Schömig et al. [44]. 

### 4.5. Zinc-Binding Affinity of Mucins

The binding affinity of commercially available porcine gastric mucins and mucins produced by the cell line HT-29-MTX were investigated with the chelating chromophore zincon [31]. Secreted mucins from HT-29-MTX were collected after culturing the cells for 13 days, washing with phosphate buffered saline (PBS) and additional incubation in DMEM without phenol red (with 100 U/mL penicillin and 100 µg/mL streptomycin, w/o FCS) for 24 h. Subsequently, the cell supernatant was collected and secreted mucins were concentrated to an 8-fold increase after desalting and washing with TBS using ultrafiltration (molecular weight cut-off: 50 kDA) followed by zinc depletion using Chelex^®^ 100 Resin (zinc content before depletion: 59.2 µg/g protein; zinc content after depletion: 4.1 µg/g protein). Protein content was analyzed using BCA assay as described [57]. Finally, zincon in TBS pH 7.5 (final concentration 50 µM) was added to either 1 mg/mL commercially available porcine mucin or the equivalent protein amount of secreted mucins from HT-29-MTX, respectively, and titrated with 0–60 µM ZnSO_4_·7H_2_O. Subsequently, the absorption was determined on a well plate reader (M200, Tecan, Switzerland) at 620 nm. The amount of mucin-bound zinc was calculated using an external calibration. Data were analyzed with GraphPad Prism software version 5.01 (GraphPad Software Inc., San Diego, CA, USA) and a non-linear regression assuming a one site-specific binding with Hill slope as a function of the zinc concentration was applied.

### 4.6. Cellular Zinc Uptake Measured by Zinpyr-1 

Short-term zinc uptake in Caco-2, HT-29 and HT-29-MTX was quantified as the increase of free zinc [nM] using the low molecular zinc probe Zinpyr-1. The concentration of free zinc was determined using the following equation of Grynkiewicz et al.

[Zinc] = Kd × [(F − F_min_)/(F_max_ − F)] [58]

and a dissociation constant for the zinc-Zinpyr-1-complex of 0.7 nM [59]. Cells were transferred to 96-well plate and cultured for 21days (Caco-2; initial cell number per well: 5000) or 7 days (HT-29 and HT-29-MTX; initial cell number per well: 10,000), respectively. On the day of the experiment, cells were incubated with 2.5 µM Zinpyr-1 and the uptake of zinc, in the presence or absence of mucins as indicated in the respective figure legends, was measured as the increase of free zinc on a fluorescence well plate reader (Spark, Tecan, Switzerland; Zinpyr-1 fluorescence: λ_ex_ = 508 nm and λ_em_ = 527 nm) as described [17].

### 4.7. Removal of Extracellular Mucins by N-Acetylcysteine 

Extracellular mucins of the goblet cell line HT-29-MTX were removed by reducing the intra- and intermolecular disulfide bounds in the glycoproteins using NAC [21,60]. Before analyzing zinc uptake of HT-29-MTX, cells were treated twice with 10 mM NAC in PBS for 10 min. Between treatments, cells were incubated in DMEM for 1 h. Successful removal of extracellular mucins was examined using immunochemical staining of the main glycoprotein of HT-29-MTX, MUC5AC, and fluorescence microscopic visualization with FD-20.

### 4.8. Immunochemical Staining of the MUC5AC Glycoprotein

Immunochemical staining of MUC5AC was performed using the mucin-specific antiserum MAN-5ACI for the polypeptides of MUC5AC [61,62]. Therefore, HT-29-MTX and HT-29 cells were seeded on glass slides and cultivated for 7 days. Depletion of extracellular mucins of HT-29-MTX prior to immunochemical staining was performed as described above. Cells were fixed on ice with a final concentration of 3.7% formaldehyde directly added to the cell medium, washed with cold PBS and permeabilized using 0.5% Triton-X-100 in PBS for 20 min on ice. After washing with PBS, cells were blocked with 10% FCS in TBS for 1 h, incubated with MAN-5ACI antiserum (1:500 in TBS with 20% Tween (TBST)) overnight, followed by additional washing and blocking. Subsequently, Cy3^®^ goat anti rabbit IgG (indocarbocyanin goat-anti rabbit immunoglobulin G) (1:500 in TBST) was incubated for 1 h at 37 °C. Additionally cellular nuclei were stained with Hoechst 33258. Finally, cells were washed with TBST and evaluated by fluorescence microscopy (Axio Imager M1, Zeiss, Germany) at excitation wavelengths of 546 nm (Cy-3) and 358 nm (Hoechst).

### 4.9. Visualizing Extracellular Mucins with Fluorescein Isothiocyanate (FITC)-Dextran 

Extracellular mucins produced by HT-29-MTX were visualized using high molecular dextran labeled with fluorescein isothiocyanate (FD-20). Due to its size, FD-20 is trapped in the high molecular glycoproteins, possibly due to steric hindrance [63]. To this end, HT-29 and HT-29-MTX cells were cultured in glass bottom dishes for 14 d. Prior to the experiment, medium was carefully removed and 100 µM FD-20 together with cell membrane tracker CellMask™DeepRed (3.3 ng/mL) in assay buffer (120 mM NaCl, 5.4 mM KCl; 5 mM Glucose; 1 mM CaCl_2_, 1 mM MgCl_2_, 1 mM NaH_2_PO_4_, 10 mM HEPES, pH 7.35) was incubated for 15 min at 37 °C. Subsequently, cells were washed with assay buffer and live cell imaging of fluorescently labeled extracellular mucins was performed with a CLSM (Leica TCS SP8; λ_ex_(FITC) = 488 nm, λ_em_(FITC) = 510 nm; λ_ex_(CellMask™DeepRed) = 552 nm, λ_em_ (CellMask™DeepRed) = 695 nm). 

### 4.10. Total Cellular Zinc Content Measured by Flame Atomic Absorption Spectrometry (FAAS)

For the determination of long-term zinc uptake with FAAS, 1.2 × 10^5^ Caco-2 cells were seeded in 6 well plates and cultured for 21 days. Fully differentiated cells were incubated with different zinc concentrations with 0 or 5 mg/mL porcine mucin in DMEM w/o phenol red and incubated for 24 h. Short-term uptake was conducted after 30 min incubation using 0 or 5 mg/mL mucin in assay buffer. Finally, cells were harvested on ice with a cell scraper, and an aliquot was collected for protein quantification as described [57]. Subsequently, cells were dissolved in a mixture of 67% ultrapure HNO_3_ and 30% H_2_O_2_ (50/50; *v*/*v*) and dried at 92 °C overnight using a thermoshaker. Residues were dissolved in 0.67% HNO_3_ and samples were analyzed by FAAS.

### 4.11. Zinc Transport Assay

Zinc transport studies were performed using monocultures of Caco-2 and Caco-2/HT-29-MTX co-cultures. Co-cultures were realized with an initial cell ratio of 75% Caco-2 and 25% HT-29-MTX cells and alternated cell seeding, modified after Nollevaux et al. [64]. Herein, the co-culture of Caco-2 and HT-29-MTX cells in our lab was characterized concerning the proper cellular ratio by investigating the mucin secretion and adequate differentiation of the enterocytes as reported before [17]. 

Eighty thousand cells were transferred onto polycarbonate transwell membranes (pore size 0.4 µm, culture area 1.12 cm^2^) and cultured for 21 days in DMEM with 10% FCS, 100 U/mL penicillin, 100 µg/mL streptomycin and 1% NEAA. For the co-cultures, 25% 20,000 HT-29-MTX cells were added 2 days after seeding of Caco-2. After 21 days, the cells were incubated with 0 µM, 25 µM, 50 µM and 100 µM ZnSO_4_·7H_2_O in 0.5 mL transport buffer [17] on the apical side of the transport chamber for 4 h. The basolateral compartment constituted 1.5 mL cell culture medium with 30 mg/mL BSA. Prior and after the experiment, barrier integrity was monitored by measuring TEER with the epithelial volt-ohm meter Millicell^®^ ERS-2 (Millipore, Burlington, MA, USA). Additionally, permeability of cell monolayers during the experiment was determined using FD-20 [64] as reported before [17]. At the end of the experiment, the media of the apical and basolateral compartments were collected, and cells were harvested on ice in PBS, homogenized and centrifuged (800× *g*). An aliquot of cell homogenates was collected for protein quantification using BCA [57]. Subsequently, cells were dried at 92 °C overnight as described above and dissolved in 0.67% HNO_3_. Zinc quantification in apical, basolateral and cellular compartment was conducted by ICP-MS after dilution (1:10 and 1:200) in 2% HNO_3_ containing 5 µg/L rhodium, using an Agilent 8800 ICP-QQQ (Agilent Technologies Deutschland GmbH, Böblingen, Germany) in the single quad-mode [17]. 

### 4.12. Statistical Analysis

Statistical significance was analyzed by one- or two-way ANOVA (for multiple comparisons), followed by Bonferroni or Dunnett’s multiple comparison post hoc tests, as indicated in the respective figure legends, using GraphPad Prism software version 5.01 (GraphPad Software Inc., San Diego, CA, USA). Error bars represent standard deviation or standard error of the mean, as indicated, of at least three independent biological replicates.

## 5. Conclusions

This study provides the first comprehensive assessment of the zinc binding properties of mucins and their impact on in vitro intestinal zinc resorption. By clarifying the molecular zinc binding capacity of these glycoproteins and their average affinity for the bivalent cation, we could demonstrate that mucins bind multiple zinc ions with physiologically relevant affinity. Hereby, zinc-free mucins impair zinc uptake, but this is not the form in which they are present in the gastrointestinal tract. 2D-experiments with isolated porcine mucins show that mucin-bound zinc is still available for cellular uptake, but not superior to free zinc. In contrast, the 3D co-culture of enterocytes and mucin-secreting goblet cells suggests that mucins even facilitate zinc uptake by enterocytes, making them an integral part of intestinal zinc resorption.

## Supplementary Materials

Supplementary materials can be found at http://www.mdpi.com/1422-0067/19/9/2662/s1.

## Abbreviations

ANOVA	analysis of variance
BCA	bicinchoninic acid
BSA	bovine serum albumin
CLSM	confocal laser scanning microscopy
DMEM	Dulbecco’s modified Eagles medium
ECACC	European Collection of Cell Cultures
HBSS	Hanks’ balanced salt solution
HEPES	4-(2-hydroxyethyl)-1-piperazineethanesulfonic acid
FAAS	flame atomic absorption spectrometry
FCS	fetal calf serum
FD	(FITC)-Dextran
FITC	fluorescein isothiocyanate
ICP-MS	inductively-coupled plasma mass spectrometry
NAC	*N*-acetylcysteine
NEAA	non-essential amino acids
PAR	4-(2-pyridylazo)resorcinol
PAS	periodic acid Schiff
Papp	apparent permeability
PBS	phosphate buffered saline
TBS	tris(hydroxymethyl)aminomethane (Tris)-buffered saline
TBST	tris-buffered saline with Tween 20
TEER	transepithelial electrical resistance
TPEN	*N*,*N*,*N*′,*N*′-Tetrakis(2-pyridylmethyl)ethylenediamine
ZnT	zinc transporter
ZIP	Zrt Irt-like transporter
Zincon	2-carboxy-2′-hydroxy-5′-sulfoformazylbenzene monosodium salt
