# Supplementary material for: In Vitro Studies on Zinc Binding and Buffering by Intestinal Mucins
Source: Int J Mol Sci. 2018 Sep 7;19(9):2662. doi: 10.3390/ijms19092662 (PMC6164875; doi:10.3390/ijms19092662)
Supplement: Supplementary file 1 [file ijms-19-02662-s001.pdf]

Supplemental Material

In vitro studies on zinc binding and buffering by intestinal mucins

Maria Maares[1], Claudia Keil[1], Jenny Koza[1], Sophia Straubing[1], Tanja Schwerdtle[2, 3], Hajo Haase[1, 3*]

[1] Department of Food Chemistry and Toxicology, Berlin Institute of Technology, Germany;

[2] Institute of Nutritional Science, University of Potsdam, Germany;

[3] TraceAge - DFG Research Unit on Interactions of essential trace elements in healthy and diseased elderly, Potsdam-Berlin-Jena, Germany

*Corresponding author:

Department of Food Chemistry and Toxicology, Berlin Institute of Technology, Gustav-Meyer-Allee 25, D-13355 Berlin, Germany

Tel: +49 (0) 30 31472788

Fax: +49 (0) 30 31472823

E-Mail: Haase@TU-Berlin.de

Abbreviations:

ANOVA, analysis of variance; FD, (FITC)-Dextran; FAAS, flame atomic absorption spectrometry; FITC, Fluorescein isothiocyanate, ICP-MS, inductively-coupled plasma mass spectrometry; PAR, 4-(2-pyridylazo)resorcinol; Papp, apparent permeability; TBS (Tris(hydroxylmethyl)aminomethan buffered saline); TEER, transepithelial electrical resistance; zincon, 2-carboxy-2′-hydroxy-5′-sulfoformazylbenzene monosodium salt

A) Absorptionspectra PAR-zinc complex

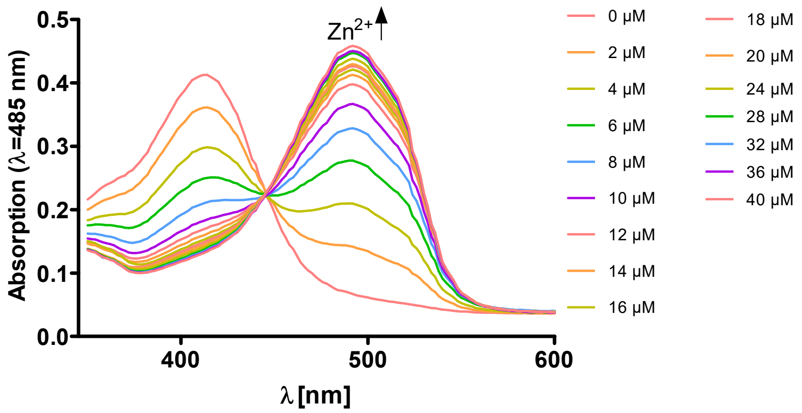

B) Spectrophotometric titration of PAR with zinc

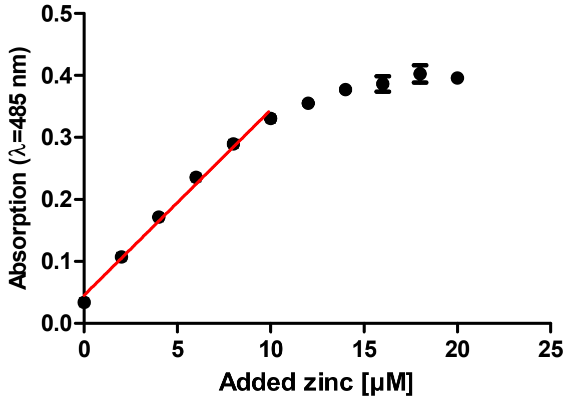

Figure S1: Spectrophotometric titration with 4-(2-pyridylazo)resorcinol (PAR) and zinc. Shown are absorption spectra (A) and spectrophotometric titrations (B) of 20 µM PAR with different zinc concentrations in Tris(hydroxylmethyl)aminomethan buffered saline (TBS), pH 7.4. Data are presented as means ± SD of three independent experiments.

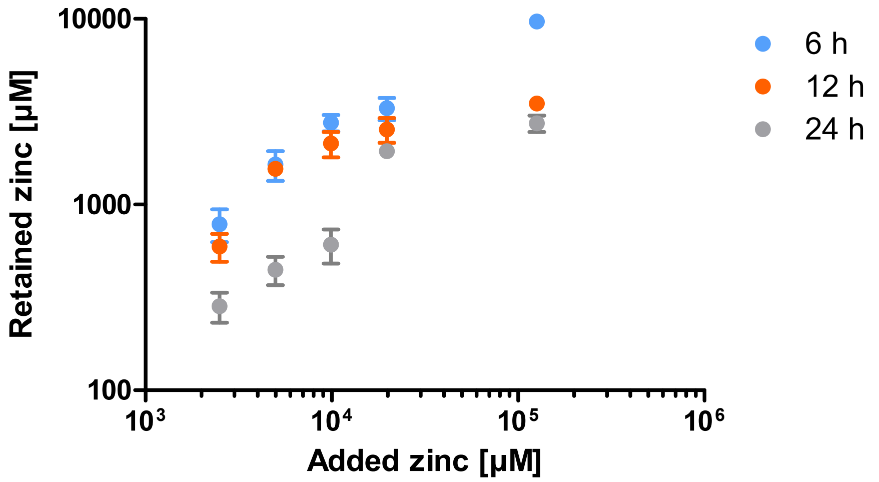

Figure S2: Zinc binding capacity of mucins using dialysis for different time intervals. The zinc binding capacity was investigated after dialysis against TBS after different time intervals. Therefore, 25 mg/mL porcine mucin was incubated overnight with different zinc concentrations and dialysis was performed for 6 h, 12 h and 24 h. The amount of zinc retained by binding to mucins was measured using flame atomic absorption spectrometry (FAAS). Data are presented as means ± SD of three independent experiments.

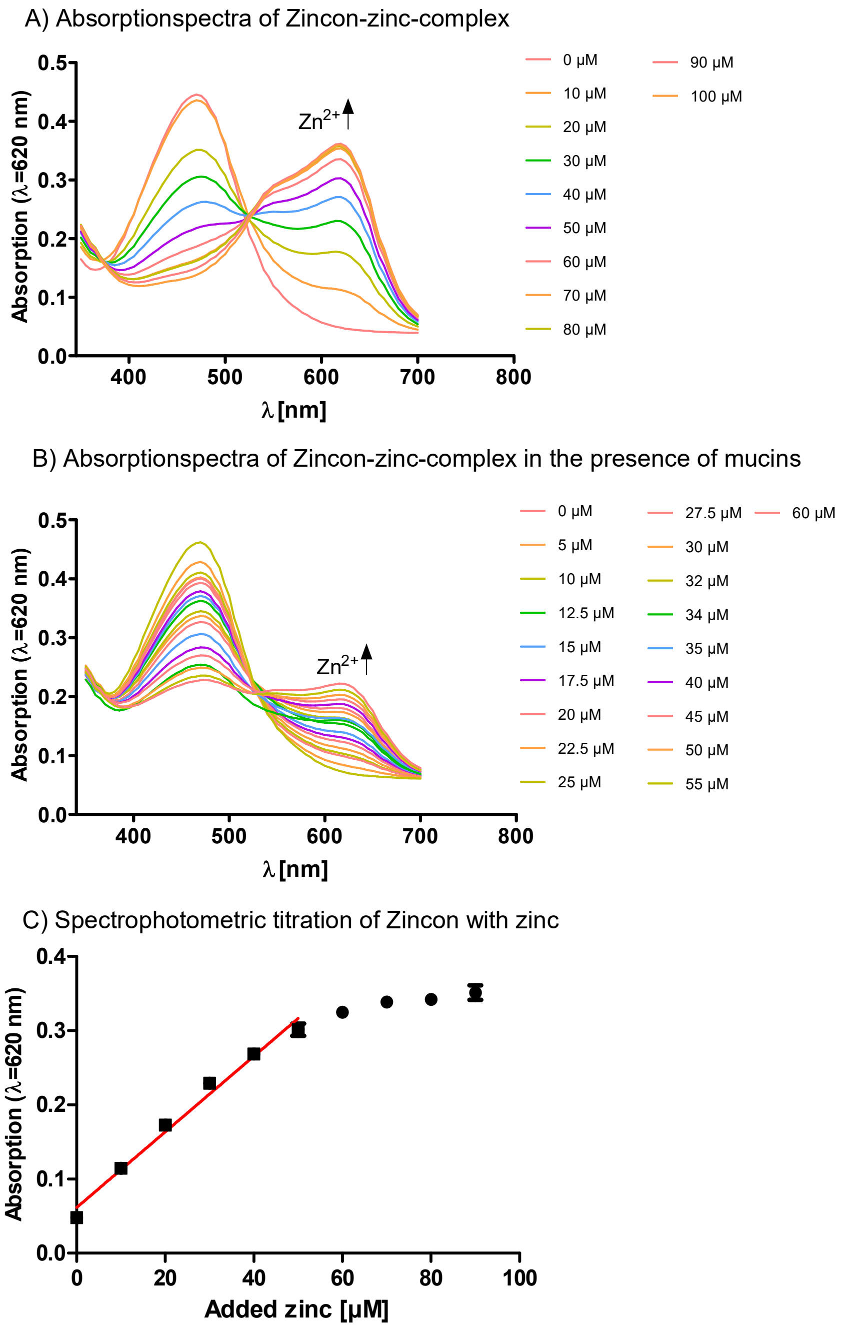

Figure S3: Spectrophotometric titration with 2-carboxy-2′-hydroxy-5′-sulfoformazylbenzene monosodium salt (zincon) and zinc. Shown are the absorption spectra (A, B) and spectrophotometric titrations (C) of 50 µM zincon with different zinc concentrations in TBS, pH 7.4. Moreover, the absorption spectrum was conducted in the presence of 0 mg/mL (A) and 1 mg/mL porcine mucins (B). Data are presented as means ± SD of three independent experiments.

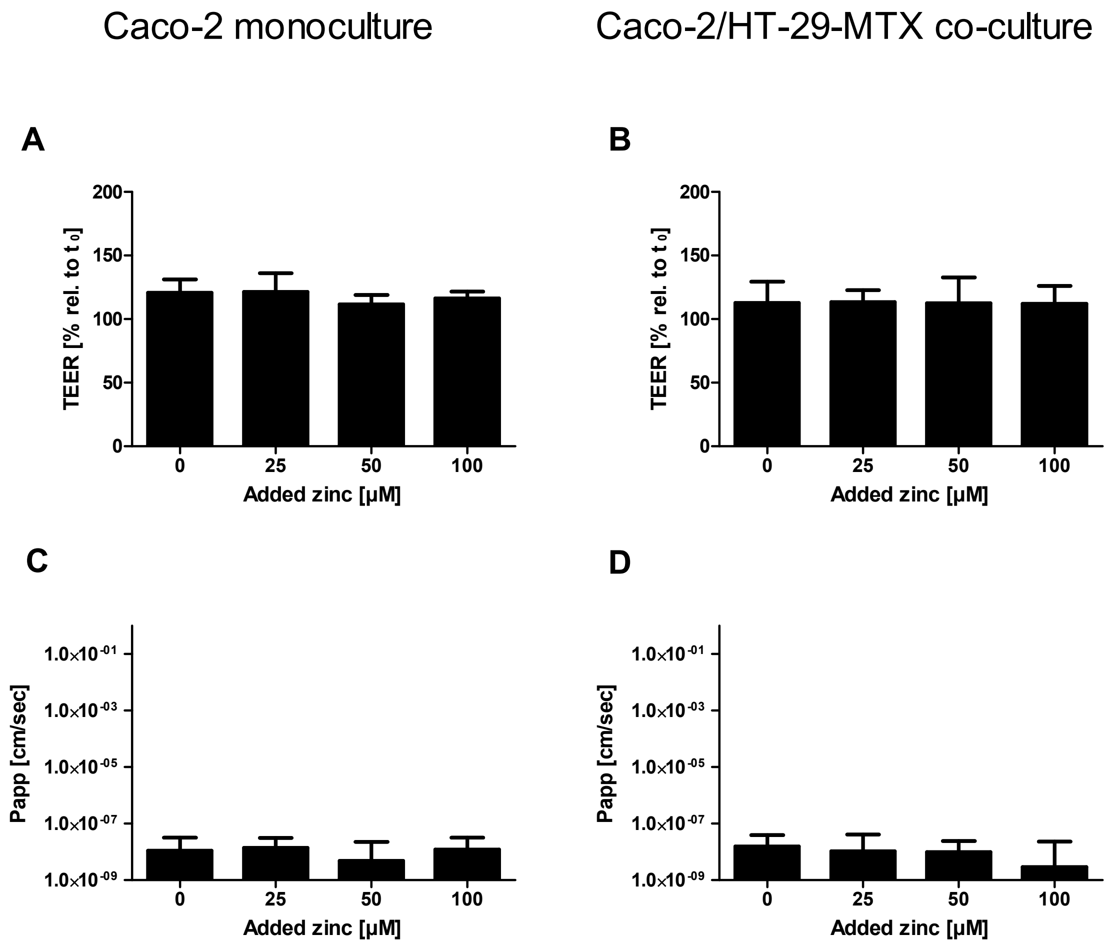

Figure S4: Integrity of Caco-2 and Caco-2/HT-29-MTX cell monolayers used for the transport studies measured as transepithelial electrical resistance (TEER) and paracellular permeability. Shown is the TEER of Caco-2 (A) or Caco-2/HT-29-MTX monolayers (B) after the transport experiment relative to TEER measured prior to incubation with zinc. The permeability of the cell monolayer during the transport assay using the monoculture (C) or the co-culture (D) is depicted as the apparent permeability (Papp) of a 20 kDa fluorescein isothiocyanate (FITC)-Dextran. Data are presented as means ± SD of three independent experiments.

	Caco-2 monocultures				Caco-2/HT-29-MTX co-cultures			
Added zinc [µM]	0	25	50	100	0	25	50	100
Apical zinc uptake [ng/cm²]	-	93.8±45.4	168.8±52.7	198.4±88.5	-	89.2±18.5	193.4±16.5	332.2±137.4
Cellular zinc [ng/cm²]	59.6±14.5	76.8±21.2	86.9±23.6	95.6±25.9	37.6±2.6	67.7±13.5	101.4±31.8	130.8±38.1
Resorbed zinc [ng/cm²]	29.9±14.5	48.8±6.5	70.8±8.9	93.3±12.5	47.0±7.5	83.7±6.4	105.1±24.7	149.6±35.1

Table S1: Exact amounts of zinc [ng/cm²] transported by Caco-2/HT-29-MTX co-cultures and Caco-2 monocultures. Shown are the amounts of zinc which are absorbed into the cells (zinc uptake), the cellular zinc content and which are resorbed into the basolateral compartment in ng zinc per resorption area (in cm²). Data are presented as means ± SD of three independent experiments.
